# Fabrication and Characterization of an Optimized Low-Loss Two-Mode Fiber for Optoacoustic Sensing

**DOI:** 10.3390/mi13101774

**Published:** 2022-10-19

**Authors:** Zelin Zhang, Guanglei You, Yu Qin, Jianqin Peng, Shuhong Xie, Xinli Jiang, Caoyuan Wang, Ruowei Yu, Yichun Shen, Limin Xiao

**Affiliations:** 1 Zhongtian Technology Advanced Materials Co., Ltd., Zhongtian Technology Group, Nantong 226010, China; 2 Advanced Fiber Devices and Systems Group, Key Laboratory of Micro and Nano Photonic Structures (MoE), Key Laboratory for Information Science of Electromagnetic Waves (MoE), Shanghai Engineering Research Center of Ultra-Precision Optical Manufacturing, School of Information Science and Technology, Fudan University, Shanghai 200433, China; 3 Zhongtian Technology Fiber Co., Ltd., Zhongtian Technology Group, Nantong 226010, China; 4 College of Physics, Nanjing University of Aeronautics and Astronautics, Nanjing 210016, China; 5 Zhongtian Technology Group, Nantong 226463, China

**Keywords:** few-mode fiber, Brillouin scattering, optoacoustic interaction, optoacoustic chemical sensing

## Abstract

An optimized multi-step index (MSI) 2-LP-mode fiber is proposed and fabricated with low propagation loss of 0.179 dB/km, low intermodal crosstalk and excellent bend resistance. We experimentally clarified the characteristics of backward Brillouin scattering (BBS) and forward Brillouin scattering (FBS) induced by radial acoustic modes (*R*_0,*m*_) in the fabricated MSI 2-LP-mode fiber, respectively. Via the use of this two-mode fiber, we demonstrated a novel discriminative measurement method of temperature and acoustic impedance based on BBS and FBS, achieving improved experimental measurement uncertainties of 0.2 °C and 0.019 kg/(s·mm^2^) for optoacoustic chemical sensing. The low propagation loss of the sensing fiber and the new measurement method based on both BBS and FBS may pave the way for long-distance and high spatial resolution distributed fiber sensors.

## 1. Introduction

Few-mode fibers (FMFs), due to their potential to raise the transmission capacity, have attracted intensive attention in optical communications and advanced sensors [1,2,3]. Combined with the space division multiplexing (SDM) technique, the FMF, as one of the key specialty fibers, is addressing the impending transportation “capacity crunch” by adding more spatial channels with several linear polarized (LP) modes within a single fiber [3,4]. In order to achieve mode division multiplexed transmission efficiently in a complex circumstance, the requirements for such an FMF should be low attenuation, low intermodal crosstalk between the LP groups of modes, low differential mode group delay (DMGD) caused by different propagation constant of the respective LP modes and good bending resistance. By controlling the Alpha value of the refractive index profile (RIP) precisely, the graded index (GI) FMFs can achieve low DMGD and low intermodal crosstalk [5,6]. However, it is difficult for the GI-FMF to maintain the transmission attenuation at a low level (<0.19 dB/km) [1].

In order to provide a promising FMF for long-haul SDM transmission, in our work, by optimizing fiber RIP design and drawing processes, we fabricate an optimized multi-step index (MSI) 2-LP-mode fiber with low fiber attenuation, low intermodal crosstalk and ultra-low bending loss. Furthermore, in order to explore the potential of the proposed FMF in Brillouin scattering-based fiber sensors, we theoretically and experimentally investigate its Brillouin gain spectrum (BGS) and forward Brillouin scattering spectrum (FBSS) induced by radial acoustic modes (*R*_0,*m*_). Furthermore, by measuring its frequency shift dependencies of BGS and *R*_0,*m*_-induced FBSS on temperature and acoustic impedance, respectively, we demonstrate this optimized MSI 2-LP-mode fiber as a new sensing fiber for optoacoustic sensing and propose a novel simultaneous measurement method of temperature and acoustic impedance based on FBS and BBS with low measurement uncertainties.

## 2. Low-Loss MSI 2-LP-Mode Fiber

**Figure 1:**
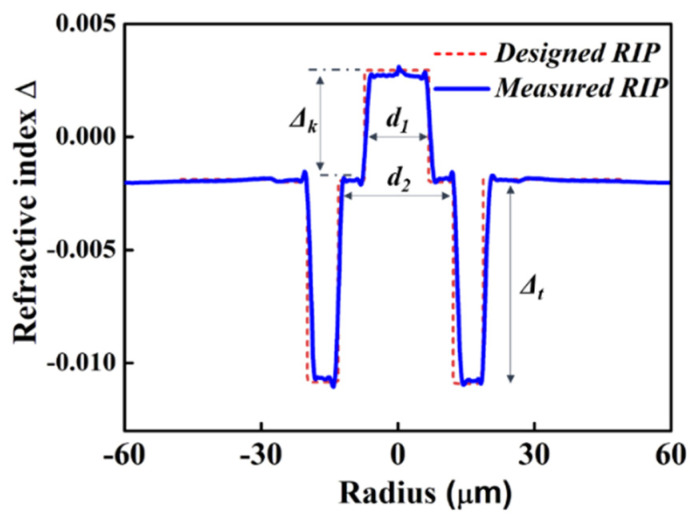
Designed and measured refractive index profile of MSI 2-LP-mode fiber.

A trench-assisted multi-step designed refractive index profile (RIP) of the 2-LP-mode fiber is shown in Figure 1, which consists of a central core with a high refractive index embedded in the large core with a low refractive index and a refractive index trench around the whole fiber core. The design strategies of our optimized FMF are applied from refs. [3,6]. The DMGD and effective index difference (*δ_neff_*) between the fundamental (LP_01_) and first higher-order mode (LP_11_) can be controlled by adjusting the Δ*_k_* value precisely. By depressing the *d*_2_/*d*_1_ ratio properly, the effective area (*A_eff_*) can be increased. Next, the trench volume (Δ*_t_*) can be optimized to minimize the bending loss for these two guided LP modes while maintaining high losses for the higher-order leaky LP modes to ensure effective cut-off. Finally, an optimized RIP is obtained, where the values of Δ*_k_*, Δ*_t_* and *d*_2_/*d*_1_ are 4.2 × 10^−3^, 9.1 × 10^−3^ and 1.8, respectively.

The preform of 2-LP-mode fiber is developed with MCVD to match the designed index profile closely. The fiber core is crafted from silica doped with germania, and the trench is made from silica with fluorine. Different from GI few-mode fibers [5,6,7,8], the drawing processes of this 2-LP-mode fiber have been optimized to obtain better geometrical uniformity and lower attenuation. The furnace is precisely controlled at a higher heating temperature of 2095 °C. Using the constant drawing tension control process, we use the lower drawing tension, which is maintained at 70 ± 3 g. The preform is drawn into the fiber under the optimized drawing speed (300 m/min). The key parameters of the MSI 2-LP-mode fiber, such as *α* (Fiber attenuantion@1550 nm), *A_eff_* (Effective mode area), *D* (Dispersion coefficient), DMGD (Differential mode group delay), *δ_neff_* (Effective index difference between LP_01_ and LP_11_ @1550 nm) and *B_L_* (Micro-bending loss @1550 nm), are shown in Table 1.

As shown in Table 1, using the optical time domain reflectometry (OTDR), the measured attenuation at 1550 nm of 0.179 dB/km is much lower than the value of 0.198 dB/km in ref. [7]. By optimizing the Δ*_k_* value, the *δ_neff_* between LP_01_ and LP_11_ can reach up to 2.6 × 10^−3^, which is two times higher than that of the two-mode fiber reported in ref. [8]. Additionally, the DMGD value can decrease to 1.9 ps∙m^−1^. In particular, the obtained MSI 2-LP-mode fiber has excellent bend resistance. When the 10 turns are introduced with the bend radius of 5 mm, the measured *B_L_* value is less than 0.006 dB at 1550 nm. Furthermore, the effective areas of LP_01_ and LP_11_ are both above 130 μm^2^ in Table 1, which make the fiber suitable for increasing the nonlinear thresholds for long-haul transmission [9].

## 3. BBS and FBS Effects in MSI 2-LP-Mode Fiber

As shown in Table 1, the optimized MSI 2-LP-mode fiber, due to its low fiber attenuation, low intermodal crosstalk and low bending loss, can act as a good candidate for long-haul optical communication. Meanwhile, its capacities based on Brillouin scattering are also desirable to be investigated in the fields of optical sensors. As a result, we will explore the BBS and FBS processes, respectively, in the MSI 2-LP-mode fiber and demonstrate its potential application in Brillouin scattering-based optical sensors.

### 3.1. BBS Effect in MSI 2-LP-Mode Fiber

**Figure 2:**
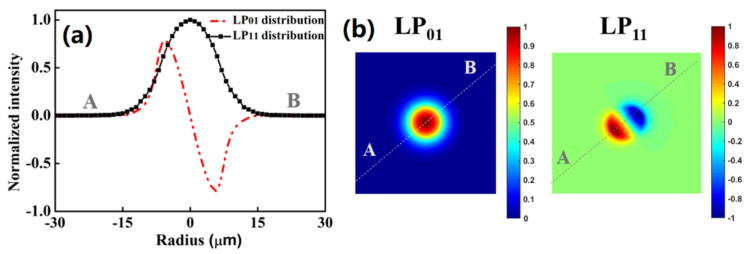
Calculated electric fields of guided LP mode LP_01_ and LP_11_ at 1550 nm in the optimized MSI 2-LP-mode fiber. (**a**) Two-dimensional mode profiles, and (**b**) electric field mode pattern.

The BBS in optical fibers is a nonlinear process where a propagating optical wave can interact with longitude acoustic waves to produce backscattered waves [10]. The BBS process can be described using the following 2D scalar equation group [9]

(1) $$\nabla^{2}E(x,y)+{\left(\frac{2\pi }{\lambda }\right)}^{2}(n^{2}-n_{eff}^{2})E(x,y)=0,$$

(2) $$\nabla^{2}u(x,y)+(\frac{\omega_{a}^{2}}{V_{L}^{2}}-\beta_{a}^{2})u(x,y)=0,$$

where *E* (*x*, *y*) and *u* (*x*, *y*) are the electric field of optical mode and the displacement field of acoustic modes, respectively. The parameter *λ* is the wavelength of the optical mode in a vacuum. Additionally, *ω_a_*, *V_L_* and *β_a_* are the angular frequency, the velocity of longitudinal acoustic mode and the propagation constant of longitude acoustic modes, respectively. Using the finite element analysis (FEA) method and solving Equation (1), the electric field distributions at 1550 nm of two LP modes supported by the MSI 2-LP-mode fiber can be theoretically obtained, which are shown in Figure 2. The two-dimensional mode profiles of two LP modes in the direction of A to B are both shown in Figure 2b.

**Figure 3:**
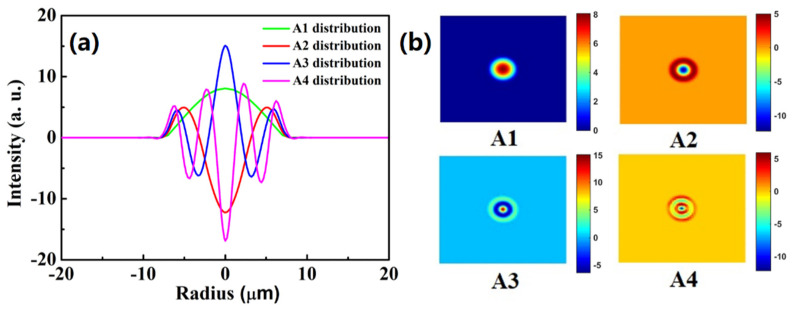
(**a**) Two-dimensional (2D) mode profiles and (**b**) field patterns of four acoustic modes.

By solving Equations (1) and (2), the four acoustic modes, named A1, A2, A3 and A4, are calculated and shown in Figure 3. It can be found that these four acoustic modes are circular symmetrical and confined in the fiber core. The higher-order acoustic modes, A2, A3 and A4, may derive from the sharp rise and fall of the index profile of 2-LP-mode fiber between the center core and the edge of the larger core [11].

When several acoustic modes are excited in the fiber simultaneously, each acoustic mode is responsible for a spectral feature in the Brillouin gain spectrum (BGS). The BGS corresponding to the *i*th acoustic mode can be expressed as [12]

(3) $$g_{i}(\nu )=g_{i}\cdot \frac{{\left(\Delta \nu /2\right)}^{2}}{{\left(v-v_{i}\right)}^{2}+{\left(\Delta \nu /2\right)}^{2}}$$

(4) $$\nu_{i}=\frac{2n_{eff}V_{eff}^{i}}{\lambda }$$

where Δ*ν*, *ν_i_* and *g_i_* are the full width at half maximum (FWHM), central frequency shift and peak Brillouin gain of the *i*th acoustic mode-induced BGS, respectively. Additionally, $V_{eff}^{i}$ is the effective acoustic velocity of the *i*th acoustic mode.

According to Equation (3), each acoustic mode-induced BGS has a Lorentzian shape. The total BGS is the sum of the BGSs of individual acoustic modes, which can be expressed as [13]

(5) $$g_{B}(\nu )=\sum_{i}g_{i}(\nu )$$

**Figure 4:**
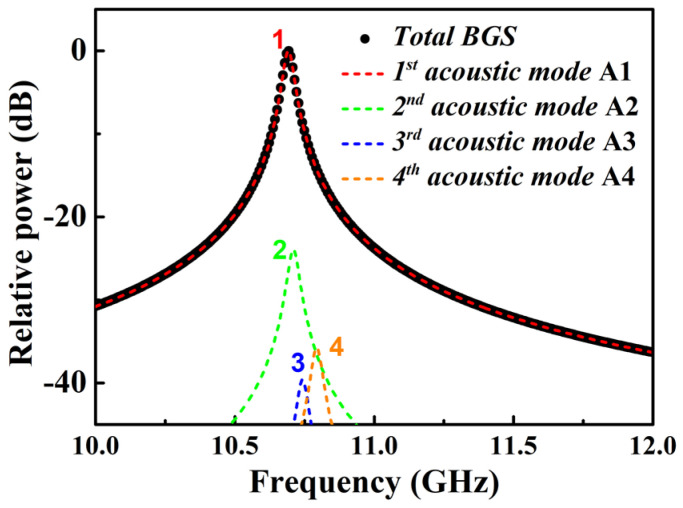
Numerical results of BGS of the MSI 2-LP-mode fiber.

By solving Equations (1)–(5), the obtained numerical BGS of the optimized MSI 2-LP-mode fiber is shown in Figure 4. It can be observed that the intensity of the first peak is more than 23 dB higher than that of the second peak. The intensities of the third and fourth peak are both less than −35 dB. As shown in Figure 2 and Figure 3, the highest intensity of the first peak can be explained by the fact that the field distribution of A1 acoustic mode tends to concentrate more towards to fiber core, which can strengthen the optoacoustic interaction between A1 and LP01 modes. The second, third and fourth Brillouin resonance peaks are so weak that the total BGS of the optimized MSI 2-LP-mode fiber has a single-peak Lorentzian shape. Furthermore, it can be calculated that the Brillouin frequency shift (BFS) and FWHM of the main peak are 10.712 GHz and 31 MHz, respectively.

### 3.2. FBS Effect in MSI 2-LP-Mode Fiber

As one of the typical optoacoustic interactions in optical fiber, the FBS process is caused by transverse acoustic modes, which involve radial acoustic modes (*R*_0,*m*_) and mixed torsional–radial acoustic modes (*TR*_2,*m*_), different from BBS induced by longitudinal acoustic waves [14]. However, due to the random variation of polarization states of the beam propagating along the non-polarization-maintaining fiber, the FBS induced by *TR*_2,*m*_ modes is much weaker than that induced by *R*_0,*m*_ modes [15]. Therefore, the FBS characteristics of the MSI 2-LP-mode fiber corresponding to the *R*_0,*m*_ modes are investigated.

Driven by the electrostriction effect in fibers [16], the *R*_0,*m*_ modes are excited, which gives rise to spatiotemporal changes in the refractive index. Due to the photo-elastic effect, these changes in the refractive index can induce the pure phase modulation to the optical wave and produce the forward-scattered stokes wave [15]. Therefore, the total FBS process can be described as the coupling amplitude equations between the optical field of optical wave ***E*** (***r***, *z*, *t*) and acoustic wave for the displacement vector ***U*** (***r***, *z*, *t*) [16,17]

(6) $$\nabla^{2}E-\frac{n_{eff}^{2}}{c^{2}}\frac{\partial^{2}E}{\partial t^{2}}=\frac{1}{\varepsilon_{0}c^{2}}\frac{\partial^{2}P_{NL}}{\partial t^{2}},$$

(7) ∂2U∂t2+Γ∂U∂t+(ηVL)2∇×(∇×U)−VL2∇(∇·U)=Fρ0,

where ***P**_NL_*, *ε*_0_, *c* and *ρ*_0_ are the total nonlinear polarization, vacuum permittivity, light velocity in vacuum and density of fused silica, respectively. The parameter *η* is the ratio between shear sound velocity and longitudinal sound velocity, and Γ is the acoustic damping parameter. F=ε0[12·γ12∇(E·E)+γ44(E·∇)E] is the electrostrictive driving term, where *γ*_12_ and *γ*_44_ are both the elements of the electrostrictive tensor for fused silica. By solving Equations (6) and (7), the normalized displacement distribution *U_m_*(*r*) of *R*_0,*m*_ mode in the MSI 2-LP-mode fiber can be calculated as

**Figure 5:**
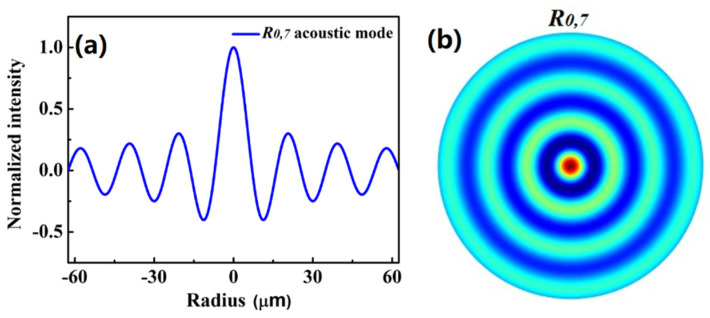
(**a**) The density vibration profile and (**b**) spatial distribution of *R*_0,7_ mode.

(8) Um(r)=J1[νfr(2πVL)]2π∫{J1[νfr(2πVL)]}2rdr

where *J*_1_ is the first-order Bessel function. The resonance frequency *ν_f_* of *R*_0,*m*_ mode can be expressed as [18]

(9) $$\nu_{f}=\frac{h_{m}V_{L}}{2\pi r_{a}}$$

where *h_m_* is the eigenvalue of *R*_0,*m*_ mode under the boundary condition corresponding to the free fiber surface, and *r_a_* is the radius of fiber cladding. Using $\rho_{m}(r)=-\rho_{0}\nabla U_{m}(r)$, the density vibration of *R*_0,*m*_ modes also can be obtained. Taking the *R*_0,7_ mode as example, the density vibration of the *R*_0,7_ mode and spatial distribution is shown in Figure 5.

**Figure 6:**
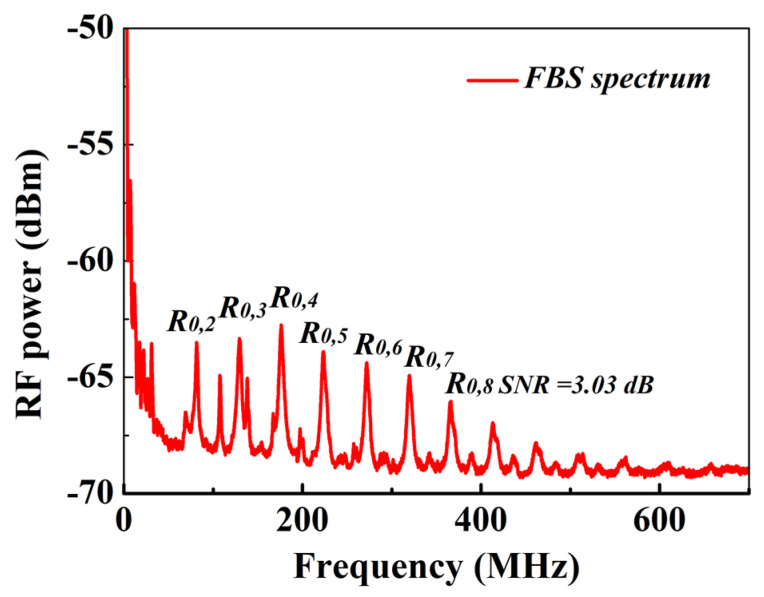
The measured FBS spectrum (FBSS) in the MSI 2-LP-mode fiber.

We also experimentally measure the FBSS of the MSI 2-LP-mode fiber using a Sagnac loop, and the measured results are shown in Figure 6. It can be found that the resonance frequencies of *R*_0,*m*_ modes in the MSI 2-LP-mode fiber are equally spaced with a spacing of 47.03 MHz. By solving Equations (6), (7) and (9), the calculated resonance frequency of *R*_0,7_ mode is 322.7 MHz, which agrees well with the measured result of 319.7 MHz in Figure 6.

Furthermore, compared with the BGS in the MSI 2-LP-mode fiber, the FBS process involves multiple acoustic modes, and the FBSS shows a multi-peak shape. These characteristics may provide some possibilities for multi-parameter optical sensors based on Brillouin scattering, such as temperature, strain and acoustic impedance and so on [14,19]. Next, using the spectral dependencies of BGS and FBSS on temperature and acoustic impedance simultaneously, we experimentally demonstrate the feasibility of this optimized MSI 2-LP mode fiber as a new sensing fiber in optoacoustic chemical sensors.

## 4. Discriminative Sensing of Temperature and Acoustic Impedance by Using the MSI 2-LP-Mode Fiber

To date, temperature and acoustic impedance simultaneous measurement methods based on FBS have been reported to use the resonance frequencies of two *R*_0,*m*_-induced FBS spectra [14] or the linewidth and resonance frequency of one *R*_0,*m*_-induced FBSS [20]. However, due to the limited spatial resolutions and sensing distances of FBS-based distributed acoustic impedance (~2 m and ≤3 km) [21,22] and temperature (~42.5 m and ≤0.4 km) [23] sensing techniques, it is not easy to achieve the medium/long-distance distributed acoustic impedance and temperature simultaneous measurement with a high spatial resolution using these two sensing methods. Here, combined with BBS and FBS effects in the MSI 2-LP-mode fiber, we propose a novel discriminative measurement method of temperature and acoustic impedance with small measurement uncertainties, which may provide the possibility to achieve longer sensing distance or higher spatial resolution by introducing BBS-based distributed mechanism for distributed acoustic impedance and temperature simultaneous measurement [24,25].

**Figure 7:**
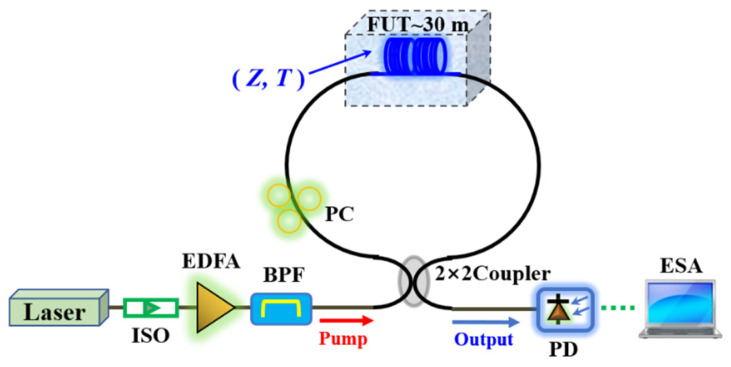
Experimental setup for observing BGS and FBSS in the MSI 2-LP-mode fiber.

We experimentally investigate the frequency shift dependencies of BGS and *R*_0,*m*_-induced FBSS on temperature and acoustic impedance in the MSI 2-LP-mode fiber, respectively. The experimental setup is shown in Figure 7. The laser source is a 1552 nm single-wavelength fiber laser with a linewidth of 15 kHz and output power of 8.2 dBm. After passing through an isolator (ISO), erbium-doped fiber amplifier (EDFA) and bandpass filter (BPF), the pump light is injected into the fiber Sagnac loop composed of a polarization controller (PC), a fiber under test (FUT) made of an uncoated 30 m-long MSI 2-LP-mode fiber and a 2 × 2 fiber coupler. In our experiment, the optical gain of EDFA is adjusted to 11.2 dB, and its noise figure is suppressed to 5 dB. The BPF has an ultra-narrow 3 dB bandwidth (<3 GHz) and high optical signal-to-noise (<50 dB) so that it can filter the amplified spontaneous emission (ASE) noise from EDFA as much as possible. The insertion loss of BPF is 2.1 dB. In the FUT, the excited *R*_0,*m*_ modes and longitude acoustic modes can both apply acousto-optic phase modulation to the propagating light [15]. Then, the fiber Sagnac interferometer can convert these two types of phase modulation into intensity modulation, which will be detected by a photo-detector (PD) with 12.5 GHz bandwidth. Finally, the lower-frequency FBS spectrum and higher-frequency BGS can be simultaneously observed and analyzed by an electrical spectrum analyzer (ESA). The frequency range of ESA is from 1 Hz to 26.5 GHz, and the spectrum is averaged 500 times.

**Figure 8:**
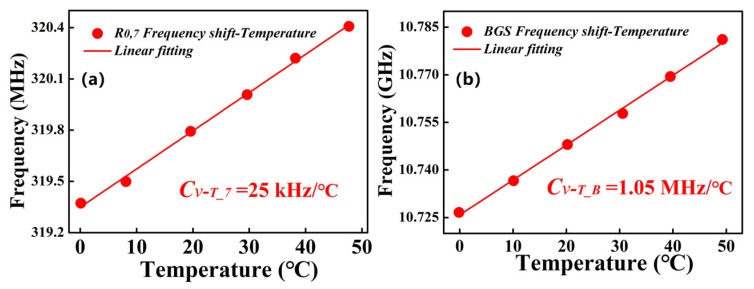
Frequency shift for (**a**) *R*_0,7_-induced FBSS and (**b**) BGS as a function of temperature.

Firstly, the MSI 2-LP-mode fiber is placed into a water bath to measure the FBS spectra and BGS as a function of temperature, respectively. As shown in Figure 6, taking consideration of the signal-to-noise ratio (SNR) and *R*_0,*m*_ modes induced FBS spectra dependencies on temperature (note that some low-order *R*_0,*m*_ modes exhibit the higher SNRs but lower frequency shift-coefficients [14]), the *R*_0,7_-induced FBSS is selected to investigate its frequency shift dependencies on temperature. As shown in Figure 8a,b, it can be clearly observed that, within the range from 0 to 50 °C, the frequency shift dependencies of *R*_0,7_-induced FBSS and BGS on temperature show the linear relationships with the different frequency shift-temperature coefficients of C*_ν-T__*_7_ = 25 kHz/°C and *C_ν-T_B_* = 1.05 MHz/°C, respectively.

**Figure 9:**
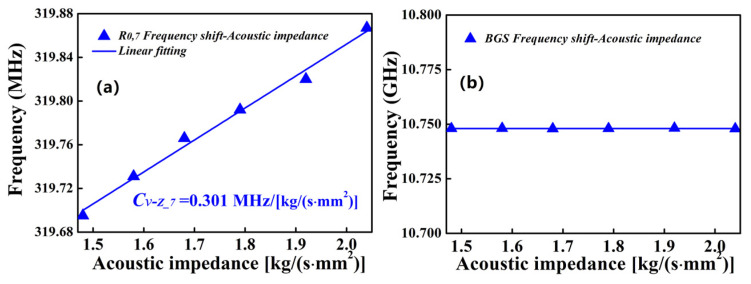
Frequency shift for (**a**) *R*_0,7_-induced FBSS and (**b**) BGS as a function of acoustic impedance.

Secondly, the FUT is immersed into the sucrose solution with different mass fractions of sucrose, which are from 0 to 50% with steps of 10%. The temperature of the sucrose solution is kept constant at room temperature (~20 °C). The relationship between the concentration of sucrose solution and acoustic impedance can be found in ref. [26]. From Figure 9a, it can be found that, with increasing acoustic impedance, the central frequency of *R*_0,7_-induced FBSS linearly shifts to high frequency with a coefficient (C*_ν-Z__*_7_) of 0.301 MHz/[kg/(s∙mm^2^)]. Interestingly, a new feature can be observed that the measured central frequency of BGS remains nearly constant when the acoustic impedance is changed to 2.02 kg/(s∙mm^2^). It means that the BGS is insensitive to the changes in acoustic impedance, and the frequency shift-acoustic impedance coefficient (C*_ν-Z_B_*) can be approximately expressed as zero. As shown in Figure 3, this feature can be explained by the fact that the total longitude acoustic modes and BBS process are confined in the fiber core, which can be isolated from external matter.

Different from refs. [14,20], the abovementioned experimental results in which the impedance *R*_0,7_-induced FBSS and BGS have their individual frequency shift dependencies on temperature and acoustic impedance may provide a novel discriminative measurement method of temperature and acoustic impedance.

When the acoustic impedance and temperature are changed simultaneously, both of them can cause the frequency shifts of *R*_0,7_-induced FBSS and BGS (Δ*ν*_7_ and Δ*ν_B_*), respectively. Thus, these can be expressed as

(10) $$\Delta \nu_{7}=C_{\nu -T\_7}\cdot \Delta T+C_{\nu -Z\_7}\cdot \Delta Z$$

and

(11) $$\Delta \nu_{B}=C_{\nu -T\_B}\cdot \Delta T+C_{\nu -Z\_B}\cdot \Delta Z$$

where the Δ*T* and Δ*Z* are the changes in temperature and acoustic impedance, respectively. The measurement uncertainties of the central frequencies of *R*_0,7_-induced FBSS and BGS (*δν*_7_ and *δν_B_*) are, respectively,

(12) $$\delta \nu_{7}=C_{\nu -T\_7}\cdot \delta T+C_{\nu -Z\_7}\cdot \delta Z$$

and

(13) $$\delta \nu_{B}=C_{\nu -T\_B}\cdot \delta T+C_{\nu -Z\_B}\cdot \delta Z$$

where the *δT* and *δZ* are the measurement uncertainties of temperature and acoustic impedance. Using Equations (12) and (13), the *δT* and *δZ* can be expressed as

(14) $$\delta T=\frac{\sqrt{{\left(C_{\nu -Z\_B}\cdot \delta \nu_{F\_7}\right)}^{2}+{\left(C_{\nu -Z\_7}\cdot \delta \nu_{B}\right)}^{2}}}{\left|C_{\nu -Z\_B}C_{\nu -T\_7}-C_{\nu -T\_B}C_{\nu -Z\_7}\right|}$$

and

(15) $$\delta Z=\frac{\sqrt{{\left(C_{\nu -T\_B}\cdot \delta \nu_{F\_7}\right)}^{2}+{\left(C_{\nu -T\_7}\cdot \delta \nu_{B}\right)}^{2}}}{\left|C_{\nu -Z\_B}C_{\nu -T\_7}-C_{\nu -T\_B}C_{\nu -Z\_7}\right|}$$

**Figure 10:**
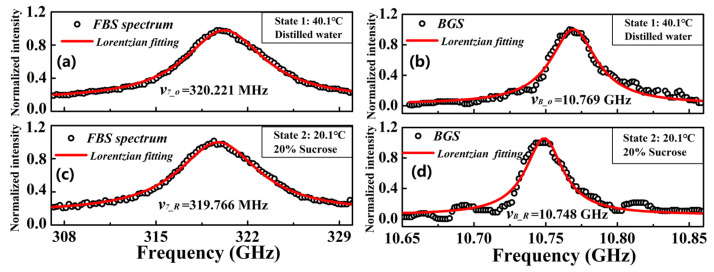
The measured induced FBSS induced by radial acoustic mode of (**a**) *R*_0,7_ at State 1 and (**c**) *R*_0,7_ at State 2 as well as BGS at (**b**) State 1 and (**d**) State 2.

In order to compare our experiment with the existing results in the literature more conveniently, the 40 °C distilled water (the corresponding acoustic impedance is 1.505 kg/(s∙mm^2^) [27]) is set as State 1, and the 20% sucrose solution (the corresponding acoustic impedance is 1.689 kg/(s∙mm^2^) [26]) is set as the State 2. The actual temperatures of these two measured states are controlled at 40.1 °C and 20.1 °C, respectively. In our experiment, the measurement uncertainties of *R*_0,7_-induced FBSS and BGS can be obtained by calculating the standard deviation of seven measured results [14,20], which are 1.6 kHz and 157 kHz, respectively. Figure 10a,c show the measured *R*_0,7_-induced FBSS at State 1 and State 2, where the corresponding central frequencies (*ν*_7___0_ and *ν*_7___*R*_) are respectively 320.211 MHz and 319.766 MHz, respectively. Figure 10b,d show the measured *R*_0,7_-induced FBSS at State 1 and State 2, where the corresponding central frequencies (*ν_B_*__0_ and *ν_B_*_*_R_*) are 10.769 GHz and 10.748 GHz, respectively.

By regarding State 1 as the original state and solving Equations (10) and (11), the temperature and acoustic impedance at State 2 can be obtained as *T_R_*_*_e_* = 19.9 °C and *Z_R_*_*_e_* = 1.670 kg/(s∙mm^2^), respectively. Therefore, the experimental measurement uncertainties of temperature (*δT_e_* = |*T_R_*_*_e_* − *T_R_*|) and acoustic impedance (*δZ_e_* = |*Z_R_*_*_e_* − *Z_R_*|) are 0.2 °C and 0.019 kg/(s·mm^2^), respectively. Moreover, by solving Equations (12)–(15), the theoretical measurement uncertainties of temperature (*δT*) and acoustic impedance (*δZ*) can be calculated as 0.15 °C and 0.014 kg/(s·mm^2^), respectively. These differences between theoretical and experimental measurement uncertainties may originate from the evaluated errors of linear coefficients. Compared with the experimental results in ref. [14], the proposed novel temperature and acoustic impedance simultaneous measurement method based on FBS and BBS in the optimized MSI 2-LP-mode fiber can achieve more than 36% accuracy improvements.

## 5. Conclusions

In conclusion, by optimizing RIP design and drawing processes, we can successfully fabricate an optimized MSI 2-LP-mode fiber with low fiber attenuation, low mode coupling and ultra-low bending loss. Then, we can theoretically and experimentally investigate the characteristics of *R*_0,*m*_-induced FBS and BGS and clarify their frequency shift dependencies on temperature and acoustic impedance in the MSI 2-LP-mode fiber. By utilizing these relationships, we have proposed and verified a novel simultaneous measurement method of temperature and acoustic impedance based on FBS and BBS. The obtained measurement uncertainties of temperature and acoustic impedance are only 0.2 °C and 0.019 kg/(s·mm^2^), respectively, achieving more than 36% accuracy improvements. In the future, combined with BBS-based distributed mechanisms, such as differential pulse-width pair [24], optical pulse coding [25] and in-line Raman amplification [28], the optimized MSI 2-LP-mode will be a new sensing fiber to achieve longer sensing distance or higher spatial resolution for distributed acoustic impedance and temperature simultaneous measurement, which would have potential applications in environmental engineering, chemical engineering and oil–gas reservoirs [14,20]. Furthermore, by employing multiple-input multiple-output (MIMO) and/or combining with a multi-core fiber structure [29,30], the proposed low-loss FMF will also be a good candidate for long-haul mode-division-multiplexing (MDM) and/or SDM to achieve a large enhancement of transmission capacity per optical fiber in the future. 

## Figures and Tables

**Table 1:** Parameters of the MSI 2-LP-mode Fiber.

Parameter	Value
*α* (dB/km)	0.179
Diameter of cladding (μm)	125.1
Diameter of coating (μm)	245.3
LP_01_ mode *A_eff_* @1550 nm (μm^2^)	133
LP_11_ mode *A_eff_* @1550 nm (μm^2^)	147
LP_01_ mode *D* (ps·nm^−1^·km^−1^)	22.8
LP_11_ mode *D* (ps·nm^−1^·km^−1^)	21.2
(LP_11_–LP_01_) *δ_neff_*@1550 nm	2.6 × 10^−3^
(LP_11_–LP_01_) DMGD (ps·m^−1^)	1.9
*B_L_* [dB, 10 turns @ R (Bending radius) = 5 mm]	≤0.006

## Data Availability

All the data presented in this study are available in this article.
